# Zika virus outbreak: ‘a perfect storm'

**DOI:** 10.1038/emi.2016.42

**Published:** 2016-03-09

**Authors:** Jing-Wen Ai, Ying Zhang, Wenhong Zhang

**Affiliations:** 1 Department of Infectious Diseases, Huashan Hospital, Fudan University, Shanghai 200040, China; 2 Department of Molecular Microbiology and Immunology, Bloomberg School of Public Health, Johns Hopkins University, Baltimore, MD 21205, USA; 3 MOH and MOE Key Laboratory of Medical Molecular Virology, Shanghai Medical College, Fudan University, Shanghai 200032, China

Zika virus, a once-overlooked single-stranded ribonucleic acid (RNA) virus that causes a mosquito-borne viral disease, is causing a major ongoing pandemic worldwide. On 1 February 2016, the World Health Organization declared Zika virus-associated clusters of microcephaly and related neurological disorders a ‘Public Health Emergency of International Concern'.^1^ Why has a largely ignored virus, which normally causes only mild symptoms, reemerged with a different pathophysiology, much like an unpredicted storm?

## THE EPIDEMIC EVOLUTION FROM SPORADIC TO PANDEMIC

Zika virus was first isolated from a *Macaca* monkey in Zika, Uganda, in 1947;^2^ human transmissions were later observed. The most important vector of Zika virus is *Aedes aegypti*.^3^ The virus can also be transmitted from mother to fetus during pregnancy or birth. Other modes of transmission such as sexual contact and transfusion with contaminated blood are also possible.

Between the 1950s and 1970s, only sporadic cases of Zika virus infection were reported in western and central Africa and south-east Asia.^4^ The disease nearly disappeared over two decades until multiple outbreaks in the Pacific islands marked the eastward movement of the virus in the 2000s. In 2007, Yap in Micronesia reported the first outbreak of Zika virus.^5^ The second outbreak occurred in 2013 in French Polynesia, with nearly 30 000 people becoming infected.^6^ From there, the epidemic gradually spread to the Cook Islands, New Caledonia and Easter Island in 2014.^7^ Genetic analyses have shown that the strains in the French Polynesia outbreak were more closely related to the Asian strains,^8^ indicating that the disease may have been introduced into the Pacific islands from Asia.

The Zika virus epidemic in South America first surfaced in February 2015, and the Ministry of Health of Brazil first confirmed the outbreak of Zika virus in northeastern Brazil in May 2015. Since then, the epidemic has spread further in South America. By 17 January 2016, the Pan American Health Organization reported that the Zika virus epidemic had spread to 18 countries or territories in the Americas.^9^

## ZIKA FEVER AND ITS POSSIBLE RELATIONSHIP WITH FETAL MICROCEPHALY

For more than half a century since its initial discovery, Zika virus seemed to pose little threat to human beings. Zika virus infection is asymptomatic in ~80% of infected individuals, and in those who develop the disease, the symptoms are usually mild and rarely result in human death.^10^ Therefore, updates on the treatment strategy for Zika virus infection have been slow, and there has been a relatively small amount of research on the Zika virus compared with other mosquito-borne viral diseases.

By the end of January 2016, nearly 4000 suspected cases of microcephaly have been reported in Brazil,^9^ constituting a 20-fold increase in incidence rate compared with the period of 2010–2014.^3^ Circumstantial evidence continues to accumulate. First, the period of the sudden microcephaly outbreak overlaps with the period when the Zika virus epidemic was introduced into Brazil.^11^ In addition, almost all mothers whose babies were diagnosed with microcephaly had symptoms of Zika virus infection in their early pregnancies.^11^ In the amniotic fluid of two mothers whose fetuses were diagnosed with microcephaly, Zika virus RNA has also been detected by reverse transcription polymerase chain reaction.^3^ However, until now, the strongest evidence may yet be the report of a case with severe brain disease and microcephaly related to vertical transmission of Zika virus.^12^ The fetal autopsy showed pathological changes detected only in the brain, suggesting a strong neurotropism of the virus.^12^

However, we are still far from drawing relevant conclusions. Currently, suspected cases are diagnosed mostly due to patients' traveling history and serological tests. Because serological tests of Zika virus have a high cross-reactivity with other flaviviruses,^3^ the epidemic scale may be exaggerated. The Brazilian outbreak affected far more people than any other previous outbreaks (Table 1), thus possibly explaining why no microcephaly cases were previously reported. However, the actual number of fetus microcephaly cases may be overestimated due to the low specificity of the screening test.^16^ Further studies on the differential diagnosis of the suspected cases are needed before we can establish a relationship between the Zika virus epidemic and fetal microcephaly.

## WHY BRAZIL?

Brazil is the center of this current outbreak, and an estimated 440 000–1 300 000 Brazilians were infected in 2015.^15^ At first glance, this outbreak of the Zika virus seemed completely random. However, several factors may have contributed to this outbreak.

First, genetic analyses have uncovered close relationships between the virus strains in the South American and Pacific Island outbreaks,^10^ suggesting that the Pacific islands may have been the springboard for the South American epidemic. Brazil held the 2014 FIFA World Cup tournament and the 2015 international canoe-racing event, which may have been the main entry routes for Zika virus into America. However, the conclusion that a small number of supposedly infected athletes or tourists entering Brazil could cause such an explosive outbreak seemed insufficient. Another assumption of the entry route of the Zika virus might have been the migration and successful implantation of mosquito reservoir hosts transported by humans via vehicles, vessels and plants. However, there is currently little evidence available, and we should conduct further studies to determine whether mosquito migration indeed contributed to this outbreak.

In addition, Brazil has a population density that is significantly higher than that of the Pacific Islands, thus making the disease much more difficult to control. Furthermore, most of Brazil has a tropical climate, and a large part of the Amazon rainforest is also located within this area, all of which could lead to a difficult battle against mosquitoes. In fact, the reported cases of Dengue fever, another arbovirus mainly transmitted by *Aedes aegypti*, increased by 227.12% in 2015 compared with 2014,^17, 18^ highlighting the poor control of *Aedes* mosquitoes in Brazil. The lack of immunity for the Zika virus in Brazilians is perhaps the most compelling factor underlying the outbreak. In a country without a history of Zika virus disease, Brazilians' total vulnerability to the virus would further accelerate disease transmission in this outbreak.

## LESSONS AND FUTURE PERSPECTIVES

The Zika virus outbreak is currently experiencing ‘a perfect storm', because of the widespread *Aedes* mosquitoes in South America, the introduction of the virus into a country with a high population density, possible viral mutations, the lack of immunity for Zika virus in Brazilians, increasing international travel and global warming all working together to create a negative dynamic with respect to human health. Currently, no country can declare itself completely safe from this outbreak, and all countries that have *Aedes* mosquitoes should stay on high alert. The Zika pandemic is still in progress, and the next few months may be crucial. Brazil is about to enter the rainy season, and the entire northern hemisphere will soon slowly transition to spring and summer, making the control of mosquitoes even more difficult. If high-population-density countries such as China, India, Japan or the United States experience a secondary outbreak (China and the United States have already reported imported cases), it would cause the Zika virus epidemic to become even more difficult to control. Currently, controlling *Aedes* mosquitoes and recommending that pregnant women avoid travel to Zika-virus-endemic regions and avoid being bitten by mosquitoes seems to be our best chance. However, in the future, we urgently need more research on the relationship between Zika virus and microcephaly, and doctors should establish differential a diagnosis of suspected cases of Zika virus infection.

The Zika virus was unexpected and caught us off guard, and but it has taught us an important lesson. How many other neglected pathogens are out there, lurking in the shadows and waiting for the next ‘perfect storm'? This is a scary question to ask. In a world with increasing globalization, international travel, urban crowding and global climate change, perhaps it is time for us to rethink our disease-control strategies, strengthen our research and public health infrastructures, and devise preventive measures against possible future disease outbreaks.

## Figures and Tables

**Table 1:** Past Zika virus outbreaks

**Country**	**Year**	**Population**	**Reported Zika virus disease**	**Estimated total cases of Zika virus disease**	**Estimated infection rate**	**Reported complications**
Yap, Micronesia^5^	2007	7391	Confirmed: 49 Probable: 59 Suspected: 72	900	75%	___
French Polynesia^3, 6^	2013	268 000	Confirmed: 396 Suspected: 8723	30 000	___	42 patients diagnosed with Guillain–Barré syndrome 32 patients had other neurological or autoimmune syndromes Unusual increase of 17 cases of central nervous system malformations in fetus and infants
Cook Islands^13^	2014	14 974	Confirmed: 50 Suspected: 932	___	___	___
New Caledonia^14^	2014	254 000	Confirmed: 1400	___	___	___
Brazil^3, 9, 15^	2015	205 338 000	___	444000–1300000	___	Around 4000 cases of microcephaly in fetus and infants (up to December 2015) 121 neurological manifestations and Guillain–Barré syndrome (up to December 2015)

## References

[bib1] World Health Organization WHO Director-General summarizes the outcome of the Emergency Committee regarding clusters of microcephaly and Guillain-Barré syndrome. Geneva: WHO, 2016. Available at http://www.who.int/mediacentre/news/statements/2016/emergency-committee-zika-microcephaly/en/ (accessed 7 February 2015)..

[bib2] Dick GW, Kitchen SF, Haddow AJ et al. Zika virus. I. Isolations and serological specificity. Trans R Soc Trop Med Hyg 1952; 46: 509–520. 12995440 10.1016/0035-9203(52)90042-4

[bib3] European Center for Disease Prevention and Control Zika Virus Epidemic in the Americas: Potential Association with Microcephaly and Guillain-Barré Syndrome. Stockholm: ECDC. 2015. Available at http://ecdc.europa.eu/en/publications/Publications/zika-virus-americas-association-with-microcephaly-rapid-risk-assessment.pdf (accessed 27 January 2016).

[bib4] World Health Organization. Zika virus outbreaks in the Americas. Wkly Epidemiol Rec 2015; 90: 609–610. 26552108

[bib5] Duffy MR, Chen TH, Hancock WT et al. Zika virus outbreak on Yap Island, Federated States of Micronesia. N Engl J Med 2009; 360: 2536–2543. 19516034 10.1056/NEJMoa0805715

[bib6] Oehler E, Watrin L, Leparc-Goffart I et al. Zika virus infection complicated by Guillain-Barré syndrome—case report, French Polynesia, December 2013. Eurosurveillance 2014; 19: pii: 20720. 10.2807/1560-7917.es2014.19.9.20720 24626205

[bib7] Musso D, Nilles EJ, Cao-Lormeau VM. Rapid spread of emerging Zika virus in the Pacific area. Clin Microbiol Infect 2014; 20: O595–O596. 24909208 10.1111/1469-0691.12707

[bib8] Buathong R, Hermann L, Thaisomboonsuk B et al. Detection of Zika virus infection in Thailand, 2012-2014. Am J Trop Med Hyg 2015; 93: 380–383. 26101272 10.4269/ajtmh.15-0022 PMC4530765

[bib9] Pan American Health Organization/World Health Organization Epidemiological Update: Neurological Syndrome, Congenital Anomalies, and Zika Virus Infection (17 January 2016). Washington, DC: PAHO/WHO. 2016. Available at http://www.paho.org/hq/index.php?option=com_docman&task=doc_view&Itemid=270&gid=32879&lang=en (accessed 28 January 2016).

[bib10] Gatherer D, Kohl A. Zika virus: a previously slow pandemic spreads rapidly through the Americas. J Gen Virol 2016; 97: 269–273. 26684466 10.1099/jgv.0.000381

[bib11] Pan American Health Organization, World Health Organization, Regional Office for the Americas Epidemiological Alert: Zika Virus Infection (7 May 2015). Washington, DC: PAHO/WHO. 2015. Available at http://www.paho.org/hq/index.php?option=com_docman&task=doc_view&Itemid=270&gid=30075&lang=en (accessed 2 February 2015).

[bib12] Miakar J, Korva M, Tul N et al. Zika virus associated with microcephaly. N Engl J Med 2016 e-pub ahead of print 10 February 2016; doi:10.1056/NEJMoa1600651. 10.1056/NEJMoa1600651 26862926

[bib13] Pyke AT, Daly MT, Cameron JN et al. Imported Zika virus infection from the Cook Islands into Australia, 2014. PLoS Curr 2014; 6 pii: ecurrents.outbreaks.4635a54dbffba2156fb2fd76dc49f65e. 10.1371/currents.outbreaks.4635a54dbffba2156fb2fd76dc49f65e PMC4055592 24944843

[bib14] Dupont-Rouzeyrol M, O'Connor O, Calvez E et al. Co-infection with Zika and dengue viruses in 2 patients, New Caledonia, 2014. Emerg Infect Dis 2015; 21: 381–382. 25625687 10.3201/eid2102.141553 PMC4313662

[bib15] Bogoch II, Brady OJ, Kraemer Mu et al. Anticipating the international spread of Zika virus from Brazil. Lancet 2016; 387: 335–336. 26777915 10.1016/S0140-6736(16)00080-5 PMC4873159

[bib16] Victora CG, Schuler-Faccini L, Matijasevich A et al. Microcephaly in Brazil: how to interpret reported numbers? Lancet 2016; 387: 621–624. 26864961 10.1016/S0140-6736(16)00273-7

[bib17] Pan American Health Organization, World Health Organization, Regional Office for the Americas 2015: Number of Reported Cases of Dengue and Severe Dengue (SD) in the Americas, by Country—January 15, 2016 (EW 52). Washington, DC: PAHO/WHO. 2016. Available at http://www.paho.org/hq/index.php?option=com_topics&view=readall&cid=3273&Itemid=40734&lang=en (accessed 8 February 2015).

[bib18] Pan American Health Organization, World Health Organization, Regional Office for the Americas 2014: Number of Reported Cases of Dengue and Severe Dengue (SD) in the Americas, by Country—March 20, 2015 (EW 53). Washington, DC: PAHO/WHO. 2015. Available at http://www.paho.org/hq/index.php?option=com_topics&view=readall&cid=3273&Itemid=40734&lang=en (accessed 8 February 2015).

